# Plant Functional Traits and Soil Nutrients Drive Divergent Symbiotic Fungal Strategies in Three Urban Street Tree Species

**DOI:** 10.3390/jof11060454

**Published:** 2025-06-14

**Authors:** Yifan Xue, Yao Wang, Jiang Shi, Jingyao Wei, Qiong Wang, Wenchen Song

**Affiliations:** 1 College of Life and Environmental Sciences, Minzu University of China, Haidian District, Beijing 100081, China; 22011887@muc.edu.com (Y.X.); wangyao2001123@126.com (Y.W.); 18586342585@163.com (J.S.); 15578775786@163.com (J.W.); 2 College of Art and Landscape/College of Forestry, Jiangxi Agricultural University, Nanchang 330045, China

**Keywords:** symbiotic microorganisms, *Sophora japonica*, *Ginkgo biloba*, *Populus tomentosa*, plant–soil–fungus interactions, urban forest ecosystem services

## Abstract

Understanding species-specific mechanisms governing symbiotic fungal responses to plant traits and soil factors is critical for optimizing urban tree “plant-soil-fungus” systems under pollution stress. To address this gap, we combined δ^13^C/δ^15^N isotope analysis and ITS sequencing for three common street trees in Beijing: *Sophora japonica*, *Ginkgo biloba*, and *Populus tomentosa*. In *S. japonica*, symbiotic fungal abundance was positively associated with leaf δ^15^N, indicating root exudate-mediated “plant-microbe” interactions during atmospheric NO_x_ assimilation. *G. biloba*, with weak NO_x_ assimilation, exhibited a negative correlation between fungal abundance and soil available N/P, suggesting mycorrhizal nutrient compensation under low fertility. *P. tomentosa* showed decreased fungal abundance with increasing soil N/P ratios and specific leaf area, reflecting carbon allocation trade-offs that limit mycorrhizal investment. These results demonstrate that symbiotic fungi respond to atmospheric and edaphic drivers in a tree species-dependent manner. Urban greening strategies should prioritize *S. japonica* for its NO_x_ mitigation potential and optimize fertilization for *G. biloba* (nutrient-sensitive fungi) and *P. tomentosa* (nutrient balance sensitivity). Strategic mixed planting of *P. tomentosa* with *S. japonica* could synergistically enhance ecosystem services through complementary resource acquisition patterns. This study provides mechanism-based strategies for optimizing urban tree management under atmospheric pollution stress.

## 1. Introduction

Urban street trees not only enhance the aesthetic appeal of cities but also contribute to carbon sequestration through photosynthesis in response to global environmental changes. They also help to mitigate air pollution from motor vehicle emissions through mechanisms such as stomatal absorption, foliar adsorption, and root–microbe interactions, thereby reducing pollutant concentrations [1,2,3]. However, existing research on street trees largely focuses on their marginal effects in reducing carbon emissions [4,5,6,7], the influence of urban canyons on pollutant dispersion [8,9], and nitrogen levels in urban rainwater, rather than nitrogen oxides from vehicle emissions [10,11]. Moreover, there is a notable gap in studies examining the impact of tailpipe emissions on subsurface ecosystems. These emissions can significantly affect belowground ecological processes, particularly mycorrhizal symbioses. For instance, areas with the highest levels of urban disturbance tend to have the lowest diversity of tussock symbiotic fungi [12]. Furthermore, studies on fungal communities associated with poplars show that the relative abundance of indicator species linked to phenanthrene pollution tends to decrease along a pollution gradient, whereas other operational taxonomic units (OTUs), such as *Cadophora*, *Alternaria*, and *Aspergillus*, increase [13]. These findings underscore the need to adopt an “above-ground and belowground linkage” perspective to gain a deeper understanding of how pollution affects subsurface ecosystems.

Within belowground systems, symbiotic fungi are key mediators of plant–environment interactions, playing a crucial role in enhancing plant stress tolerance. For example, a study reveals that root symbiotic fungi inoculation improves the drought resistance of *Pinus tabuliformis* seedlings [14]. However, most previous studies have primarily focused on the mechanisms by which symbiotic fungi enhance plant stress resistance, with limited systematic analysis of their responses to atmospheric pollution and the pathways through which they affect plants, both of which are critical for urban ecosystem services [15]. For urban ecosystem management, symbiotic fungi may function through an “atmospheric nitrogen–plant–fungus” isotope transfer chain [16]. Specifically, atmospheric nitrogen deposition alters soil nitrogen availability, which in turn affects plant root exudate composition and fungal metabolic activity. This interaction mechanism may enhance soil nutrient utilization efficiency and supports critical urban ecosystem functions. However, the processes and mechanisms underlying this effect remain poorly understood, particularly the ways in which symbiotic fungi respond to changes in plant and soil conditions. Some researchers have proposed that symbiotic fungi, such as tussock symbiotic fungi, primarily facilitate the transfer of nitrogen and phosphorus to plants, suggesting a correlation between fungal abundance and certain plant functional traits [17]. Conversely, other studies have shown that excessive soil nitrogen and phosphorus can inhibit symbiotic fungi, indicating that their abundance may be linked to both the absolute and relative levels of these nutrients in the soil [18]. Furthermore, the specific mechanisms by which symbiotic fungi interact with different tree species remain unclear. Therefore, it is essential to quantitatively investigate how fungal abundance influences plant functional traits and soil nutrient dynamics across various tree species.

As a megacity in China, Beijing features a distinctive ring-shaped road network, with street trees managed uniformly by the Beijing Municipal Bureau of Landscape Architecture and Greening (BMGGG). Its unique pollution profile and urban ecosystem make it an ideal setting for studying the interactions among atmospheric nitrogen input, plant functional traits, and soil microbial responses. Although previous research has examined the carbon sink capacity of street trees in Beijing [5], presence of heavy metal pollution [19,20,21,22], and polycyclic aromatic hydrocarbons (PAHs) in urban soils [23,24], there remains a gap in understanding the mechanisms by which symbiotic fungi respond to changes in plant and soil factors. One of the key challenges in this area of research is selecting appropriate quantitative detection methods. Although natural isotope techniques offer high precision for tracing nutrient dynamics [25], high-throughput sequencing of internal transcribed spacer (ITS) amplicons provides detailed insights into soil microbial community composition [26]. Therefore, this study adopts an innovative approach by combining δ^13^C and δ^15^N isotope labeling with microbial amplicon sequencing to investigate how soil symbiotic fungi associated with different street tree species respond to variations in plant traits and soil conditions. The findings aim to deepen our understanding of the relationship between street trees and soil symbiotic fungi, offering valuable insights for improving urban greening strategies and informing soil management planning in Beijing.

## 2. Materials and Methods

In general, the technical route of this study includes the following steps: first, we selected sampling points within the 1st to 5th ring roads of Beijing and collected air, leaf, and soil samples. The collected samples were then brought back to the laboratory for instrumental analysis. Stable isotope detection methods were applied to all three sample types (air, leaf, and soil), whereas fungal amplicon high-throughput sequencing was conducted specifically on the soil samples. The resulting data were then analyzed and processed, and structural equation modeling was used to test the observed patterns and hypotheses and then draw conclusions.

### 2.1. Sample Plots and Sampling

**Figure 1:**
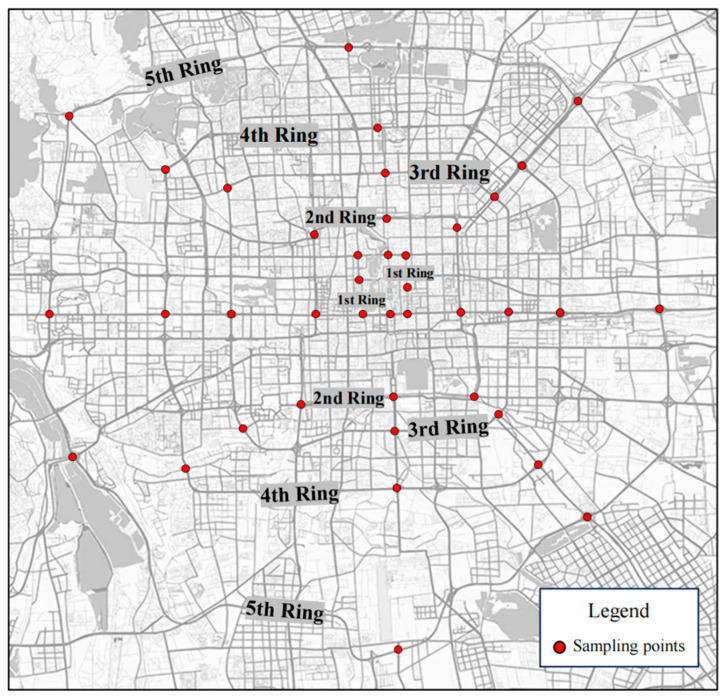
Sampling site distribution.The specific distribution of the first to fifth ring sampling points is marked with red dots in the figure.

Beijing is located between 39.4–41.66° N and 115.7–117.4° E. It has a typical radial ring city pattern, with its road network structure consisting of six ring roads (one to six ring roads) and forming a spatial layout centered around the city core and development gradually expanding outward in a gradient pattern [27]. The first to fifth ring roads are the most representative road sections. These rings reflect a hierarchical urban function layout and urbanization process, from core political and cultural functions within the first and second ring roads to a mix of commercial and residential uses along the third ring road and further to peripheral industrial and economic expansion along the fourth and fifth ring roads, illustrating a gradient change in population distribution, industrial activities, and environmental pressures as movement pushes outward from the city center [28]. In this study, the first to fifth ring roads in Beijing were used as the research object, and the strategy of concentric circle sampling and functional zoning sampling was used to evenly select eight typical sites along each ring road (Figure 1). Additionally, nine soil and leaf samples were collected from each site to systematically reflect the pollution characteristics of different ring roads and loops [29]. The study targeted three dominant tree species in Beijing: *Sophora japonica*, *Ginkgo biloba*, and *Populus tomentosa*. The sampling sites covered different directions of the ring road system, including both high-density road sections in the core urban area and emerging roads in the peripheral expansion area, thus providing comprehensive spatial distribution characteristics of road greening in Beijing [30].

The methods for air and leaf sample collection followed the procedures detailed in [30]. For soil sampling, soil was collected from the rhizosphere area of the selected sample trees using a shovel. If any of the above-mentioned tree species were present at the sample site, soil sampling was conducted for them to ensure coverage of the target tree species present at the site. Soil samples from three trees of each species were mixed into one composite sample, labeled, and placed in envelopes for analyses.

### 2.2. Sample Testing

Leaf samples were analyzed for δ^13^C, δ^15^N for isotopic composition, total carbon and nitrogen content, as well as functional traits such as leaf dry mass (LFD), specific leaf area (SLA), leaf water content (LWC), tissue density (LD), and intrinsic water-use efficiency (IWUE). Air samples were tested for δ^15^N and CO_2_ concentration. The procedures for air and leaf sample collection are described in detail in [30]. Soil samples were collected from the rhizosphere area of selected sample trees using a shovel. If any of the above-mentioned tree species were present at the sample site, soil sampling was conducted accordingly to ensure coverage of the target tree species present at the site. Soil samples from three trees of each species were mixed into one composite sample, labeled, and placed in envelopes for analyses.

Soil samples were acidified and pretreated prior to chemical analysis. Organic carbon, total nitrogen, and total phosphorus were measured using the potassium dichromate external heating method, FOSS8400 automatic Kjeldahl method (FOSS A/S, Hillerød, Denmark), and the molybdenum–antimony colorimetric method with a Lambda 25 UV spectrophotometer (PerkinElmer, Waltham, MA, USA), respectively. Available phosphorus was measured via sodium carbonate extraction and the same colorimetric assay, whereas available nitrogen was determined using the hydrazine sulfate reduction method with an AA3 continuous flow analyzer (SEAL Analytical GmbH, Norderstedt, Germany). Soil C/N and N/P ratios were calculated from the measured concentrations.

Genomic DNA was extracted from soil samples using the E.Z.N.A. Soil DNA Kit (Omega Bio-tek, Norcross, GA, USA), and DNA quality and concentration were assessed with a Nanodrop 2000 spectrophotometer (ThermoFisher Scientific, Waltham, MA, USA). The fungal internal transcribed spacer (ITS) region was amplified on an ABI 9700 PCR system (Applied Biosystems, Foster City, CA, USA) using forward primer ITS1-F (5′-CTTGGTCATTTAGAGGAAGTAA-3′) and reverse primer ITS2 (5′-TGCGTTCTTCATCGATGC-3′), and the primers were spiked with 8 bp barcode sequences. PCR was performed with 2× Taq Plus Master Mix (Vazyme Biotech Co., Nanjing, China) under the following conditions: pre-denaturation at 95 °C for 3 min, denaturation at 95 °C for 30 s, annealing at 55 °C for 30 s, extension at 72 °C for 45 s for 30 cycles, and a final extension at 72 °C for 10 min. The products were visualized using 1% agarose gel electrophoresis (170 V, 30 min), detected using a gel imaging instrument, and then purified using Agencourt AMPure XP (Beckman Coulter, Brea, CA, USA), a magnetic bead purification instrument. Libraries were prepared using the NEBNext Ultra II DNA Library Prep Kit (New England Biolabs, Ipswich, MA, USA), and the fragment sizes were screened on an Agilent 2100 Bioanalyzer (Agilent Technologies, Santa Clara, CA, USA) and sequenced using the Illumina MiSeq PE300 platform (Illumina, San Diego, CA, USA) [31].

### 2.3. Data Processing and Analysis

Raw sequencing data from the soil bioinformatics assays were processed by splitting based on the barcode sequences and then filtered and spliced using PEAR (v0.9.6) [32]. Low-quality sequences (quality value < 20), chimeras, and short sequences (<120 bp) were removed using Vsearch (v2.7.1) [33]. OTUs were clustered at 97% similarity using the UPARSE algorithm in Vsearch (v2.7.1) [34], and species annotations were assigned to each OTU against the UNITE v8.2 database using the BLAST tool(e-value ≤ 1 × 10^−5^) [35].

Functional annotation of symbiotic fungi was conducted using FUNGuild database [36], which categorizes fungal taxa into functional guilds (Symbiotroph, Saprotroph, Pathotroph, Other) based on ecological traits. Only confidently annotated symbiotic OTUs (e.g., mycorrhizal fungi and endophytes) were retained after manual verification; ambiguous or unclassified entries were excluded from downstream analyses. The relative abundance of symbiotic fungal OTUs was calculated from the OTU table using the vegan package (v2.6.8) in R. Correlations between symbiotic fungi relative abundance, plant functional traits, and soil nutrients were analyzed using dplyr (v1.1.4), and linear relationships were assessed via scatter plots with trend lines fitted using ggplot2 (v3.5.1) to determine significance. The OTU tables of the symbiotic fungi and the three trees were analyzed using Canoco 5 (Microcomputer Power, Ithaca, NY, USA). Redundancy analysis (RDA) was used to examine the relationship between plant and soil properties with significant associations. For non-significant but potentially relevant associations, a generalized additive model (GAM) was fitted using the nlme (v4.4.2) in R to capture the nonlinear relationship between the independent and dependent variables and investigate the atmosphere–plant–soil interaction mechanism. Finally, structural equation modeling (SEM) of atmospheric pollutant indicators–plant, traits–symbiotic fungi, and soil nutrients–symbiotic fungi was conducted using lavaan (v4.4.2) to quantify the role of atmospheric pollutant, plants, and soils in explaining the relative abundance of symbiotic fungi.

## 3. Results

### 3.1. Atmospheric and Plant Effects on Symbiotic Fungi

**Figure 2:**
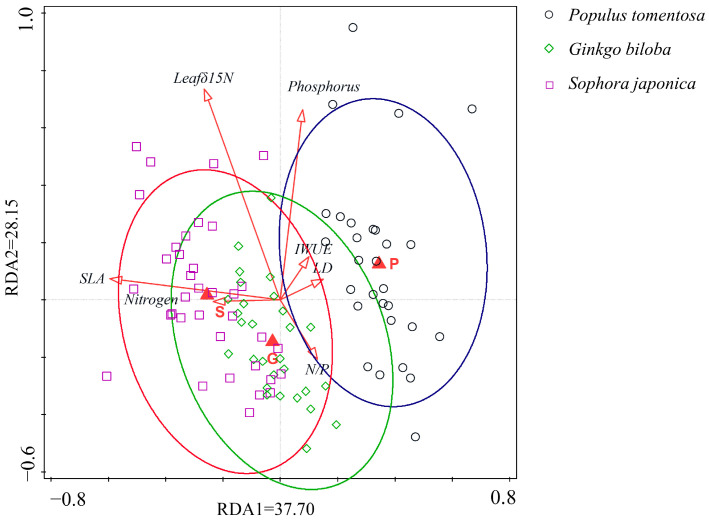
RDA analysis for three street trees (S: *Sophora japonica*; G: *Ginkgo biloba*; P: *Populus tomentosa*) plotted against plant functional traits (SLA; IWUE; LD; leaf δ^15^N) and soil nutrient factors (Nitrogen; Phosphorus; N/P).The green circles are clusters of *Ginkgo biloba*, the red circles are clusters of *Sophora japonica*, and the dark blue circles are clusters of *Populus tomentosa*.

Redundancy analysis revealed significant correlations between inter-root symbiotic fungal communities and environmental factors across the three tree species, *S. japonica*, *G. biloba*, and *P. tomentosa*, with significant species–environmental relationships and clear clustering (Figure 2). The first two RDA axes explained 37.70% and 28.15% of the variation, respectively, with a cumulative explanatory rate of 65.85%. This indicates that the plant traits (SLA; IWUE; LD; leaf δ^15^N) and soil nutrients (available and relative soil nitrogen and phosphorus content) were key drivers of the fungi community structure, with SLA showing the strongest explanatory power among the plant functional traits.

**Figure 3:**
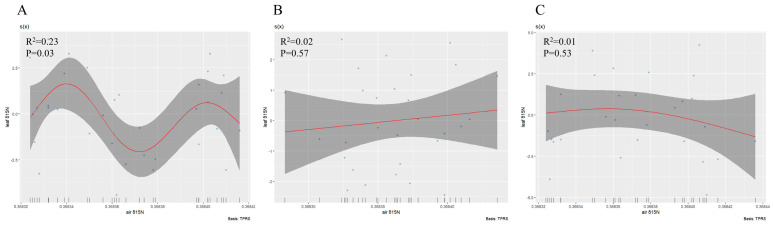
Generalized additive model fitting the relationship between atmospheric δ^15^N and leaf δ^15^N; (**A**): *Sophora japonica*, (**B**): *Ginkgo biloba*, (**C**): *Populus tomentosa*.

The generalized additive fitting model showed that leaf δ^15^N in *S. japonica* exhibited a significant nonlinear trend of initially increasing, then decreasing, and increasing again in response to atmospheric δ^15^N, whereas no significant correlations were observed for *P. tomentosa* or *G. biloba* (Figure 3A–C).

**Figure 4:**
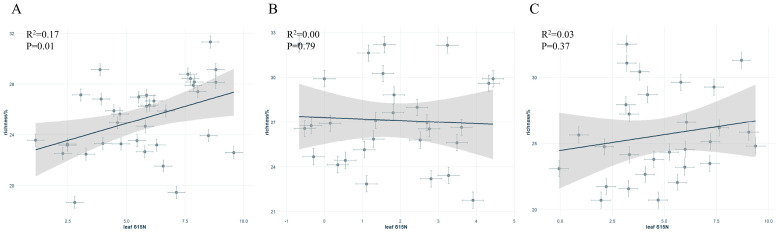
Relationship between leaf δ^15^N and symbiotic fungal abundance of different street trees: (**A**): *Sophora japonica*, (**B**): *Ginkgo biloba*, (**C**): *Populus tomentosa*.

In the scatter plot of leaf δ^15^N versus symbiotic fungi relative abundance, *S. japonica* showed a significant positive correlation, whereas no significant correlations were found for *P. tomentosa* or *G. biloba* (Figure 4A–C).

**Figure 5:**
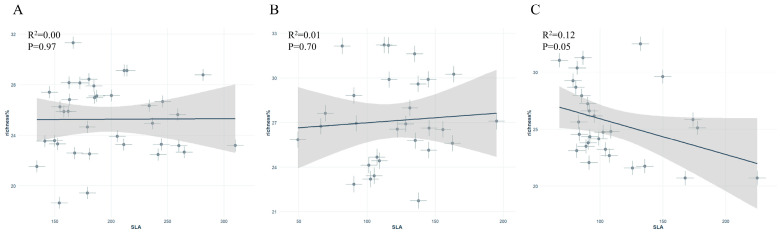
Relationship between specific leaf area (SLA) and symbiotic fungal abundance in different street trees: (**A**): *Sophora japonica*, (**B**): *Ginkgo biloba*, (**C**): *Populus tomentosa*.

Additionally, in the SLA versus symbiotic fungi relative abundance scatter plot, *P. tomentosa* showed a significant negative correlation, whereas *S. japonica* and *G. biloba* showed no significant correlations (Figure 5A–C).

### 3.2. Effect of Soil on Symbiotic Fungi

**Figure 6:**
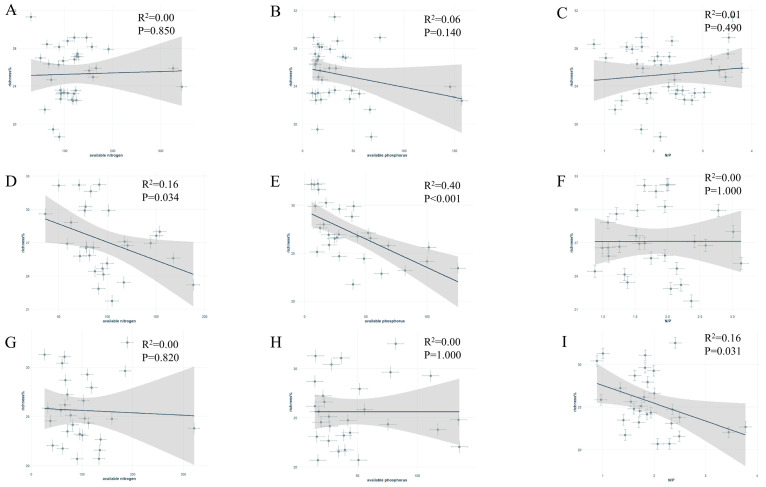
Scatter plots showing the relationship between soil nutrient factors and symbiotic fungal abundance for different street trees: (**A**–**C**): effective nitrogen, effective phosphorus, and N/P ratio versus symbiotic fungal relative abundance for *Sophora japonica*; (**D**–**F**): effective nitrogen, effective phosphorus, and N/P ratio versus symbiotic fungal relative abundance for *Ginkgo biloba*; (**G**–**I**): effective nitrogen, effective phosphorus, and N/P ratio versus symbiotic fungal relative abundance for *Populus tomentosa*.

RDA analysis showed that the available phosphorus, available nitrogen, and N/P ratio were key explanatory variables (Figure 2). The relative abundance of symbiotic fungi was influenced by these nutrient factors and varied significantly among tree species (Figure 6). In *G. biloba*, symbiotic fungal relative abundance was significantly negatively correlated with soil available nitrogen and phosphorus contents, whereas in *P. tomentosa*, it was significantly negatively correlated with the soil nitrogen–phosphorus ratio (Figure 6A–C). In contrast, *S. japonica* showed no significant correlation with both the available and relative soil nitrogen and phosphorus contents (Figure 6D–F), and the relative abundance of symbiotic fungi in *G. biloba* and *P. tomentosa* was not significantly correlated with the remaining soil nutrient contents (Figure 6G–I).

### 3.3. Structural Equation Modeling

**Figure 7:**
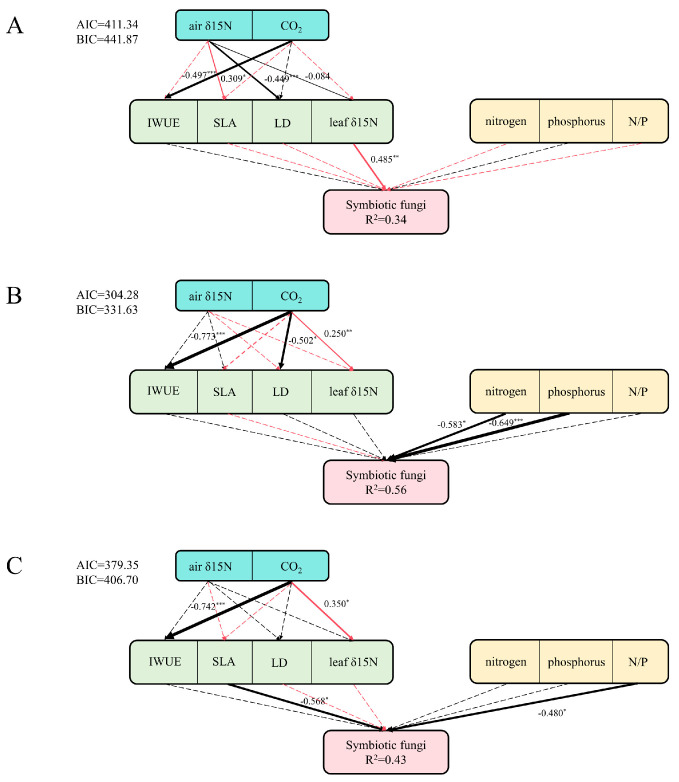
Atmosphere–plant–soil SEM modeling plots; (**A**): *Sophora japonica*, (**B**): *Ginkgo biloba*, (**C**): *Populus tomentosa*. In statistical analysis, one asterisk (*) represents a *p*-value less than 0.05, two asterisks (**) indicate a *p*-value less than 0.01, and three asterisks (***) signify a *p*-value less than 0.001.

Structural equation modeling revealed species-specific pathways linking atmospheric pollutants, plant functional traits, soil nutrients, and symbiotic fungal abundance (Figure 7). In *S. japonica*, an elevated CO_2_ concentration had a significant negative direct effect on the IWUE, whereas an elevated atmospheric NO_x_ content significantly increased leaf SLA and decreased LD. Leaf δ^15^N showed a nonlinear relationship with atmospheric NO_x_. The relative abundance of symbiotic fungi was significantly and positively influenced by the leaf δ^15^N content, with the model explaining 34% of the variation (Figure 7A). In *G. biloba*, an elevated CO_2_ concentration exhibited a significant negative effect on IWUE but positive effect on the leaf δ^15^N content. The relative abundance of symbiotic fungi was significantly negatively regulated by the soil available nitrogen and phosphorus content, with a total model explanation of 56% (Figure 7B). In *P. tomentosa*, an elevated CO_2_ concentration significantly reduced the IWUE and increased the leaf δ^15^N content. The relative abundance of symbiotic fungi was significantly and negatively affected by the plant SLA and soil N/P ratio, with a total model explanation of 43% (Figure 7C).

## 4. Discussion

### 4.1. Patterns of Atmosphere–Plant Interactions of Symbiotic Fungi of Sophora Japonica

The relative abundance of symbiotic fungi in the rhizosphere soil of *S. japonica* was predominantly influenced by plant traits, particularly leaf δ^15^N, which showed a significant positive correlation. This suggests a cascade of plant–soil–microbe interactions induced by root secretions; that is, in NOₓ-polluted environments, nitrogen-sufficient plants may allocate more carbon to the root system and provide carbon sources for fungi through the secretion of amino acids (e.g., glutamine) and carbohydrates (e.g., sucrose) [37,38], which is manifested by the significant positive correlation between δ^15^N in *S. japonica* leaves and the abundance of symbiotic fungi.

*S. japonica* accumulates a high nitrogen content owing to its efficient capacity for atmospheric nitrogen oxide (NO_x_) absorption and assimilation [39]. Its nitrogen uptake is sourced mainly from the soil, with traffic-derived NO_x_ and dry deposition contributing approximately 20% [40]. Although elevated atmospheric CO_2_ reduces the IWUE via fertilization effects [41], this process operates independently of the NO_x_–fungal pathway. Atmospheric NO_x_ exerts a dominant influence on the functional traits of *S. japonica* leaves [30]. Elevated NO_x_ concentrations enhance the stomatal uptake of gaseous nitrogen, consequently augmenting the capacity of the plant to absorb and assimilate atmospheric NO_x_.

As the δ^15^N values in anthropogenic NO_x_ emissions, such as gasoline and diesel, are typically below zero, δ^15^N in various plant parts is higher than that in NO_x_ sources, whereas soil δ^15^N frequently exceeds plant δ^15^N in unfertilized ecosystems [40]. The δ^15^N fluctuations observed in the generalized additive model analysis probably reflect the assimilation and internal transport of atmospheric pollutants in the plant. As the atmospheric NO_x_ concentrations rise, *S. japonica* exhibits distinct physiological phases: rapid absorption of gaseous nitrogen, downward transport of organic nitrogen, and nitrogen-saturated leaf stagnation. (1) During rapid absorption of gaseous nitrogen, gaseous nitrogen is rapidly absorbed through the stomata [42], where NO_2_ dissolves in the aqueous solution in the interstitial space of the leaf pulp cells and forms NO_3_⁻ and NO_2_⁻, which then enter the cytoplasm via NRT transporters [43]. (2) During downward transport of organic nitrogen, amino acids are synthesized in source leaves and transported via phloem to developing organs [44]. (3) During nitrogen-saturated leaf stagnation, excess nitrate is sequestered in vacuoles to prevent cytosolic toxicity, whereas ammonium assimilation is prioritized into amino acids [45]. This process results in a decreasing gradient between leaf δ^15^N and atmospheric δ^15^N.

In conclusion, although the functional traits of *S. japonica* responded significantly to atmospheric CO_2_ and NO_x_, the symbiotic fungi abundance was only significantly associated with leaf δ^15^N, with no significant effect from soil nitrogen. The assimilation of atmospheric NO_x_ by *S. japonica* affected leaf δ^15^N and disrupted the nitrogen balance of the plant, indirectly promoting the abundance of symbiotic fungi. Therefore, the presence of symbiotic fungi is primarily linked to the absorption and assimilation of atmospheric NO_x_ by *S. japonica*, demonstrating a clear cascade effect through plant–microbe interactions.

### 4.2. Ginkgo Biloba Symbiotic Fungi of Soil Nutrient Mutualism Patterns

The relative abundance of symbiotic fungi in soil surrounding *G. biloba* was primarily influenced by the negative correlation between the soil available nitrogen and phosphorus. Unlike *S. japonica*, *G. biloba* exhibits limited capacity for NO_x_ uptake, with its functional traits mainly influenced by CO_2_ concentrations from traffic emissions [30]. Similar to *S. japonica* [40], elevated atmospheric CO_2_ enhanced the intrinsic water-use efficiency (IWUE) in *G. biloba*. Concurrently, the leaf δ^15^N values showed a significant positive correlation with the atmospheric CO_2_ concentration, reflecting the resilience of the species to atmospheric pollution [30]. When traffic-derived CO_2_ increases in regional atmospheres, *G. biloba* enhances CO_2_ assimilation efficiency and activates antioxidant defenses via coordinated carbon–nitrogen metabolism, leading to consistent shifts in leaf δ¹⁵N isotopic signatures [46].

The negative correlation between the symbiotic fungal abundance and soil available nitrogen and phosphorus suggests a compensatory role of mycorrhizal networks under nutrient limitation. With restricted NO_x_ uptake, *G. biloba* relies on exogenous symbiotic fungi to expand nutrient absorption. These fungi facilitate the decomposition of organic nitrogen (e.g., peptides, proteins) into plant-available amino acids through extracellular enzyme secretion [47]. As a relic species that evolved during the high-CO_2_ Mesozoic era, *G. biloba* exhibits adaptive morphological traits [48]. Elevated CO_2_ further amplifies plant phosphorus demand [49], prompting *G. biloba* to employ a symbiosis-dependent strategy under phosphorus deficiency to release root secretions (e.g., citric acid) to mobilize phosphorus and selectively recruit beneficial symbiotic fungi but suppress pathogenic fungi (e.g., *Fusarium oxysporum*) [50]. These symbionts enhance phosphorus uptake via acid phosphatase (ACP) secretion [51].

In conclusion, limited atmospheric NO_x_ assimilation renders soil symbiotic fungal abundance in *G. biloba* unresponsive to atmosphere–plant interactions, but instead shows a significant negative correlation with soil available nutrients. Under nutrient-poor conditions, *G. biloba* increasingly relies on mycorrhizal networks to decompose organic nutrients and improve uptake efficiency, indicating a compensatory strategy shaped by its evolutionary history and physiological constraints.

### 4.3. Soil–Plant Co-Impact Model of Symbiotic Fungi of Populus Tomentosa

The relative abundance of symbiotic fungi in *P. tomentosa* soils is influenced by both plant functional traits and soil nutrient composition. *P. tomentosa* exhibits limited ability to assimilate atmospheric NO_x_ [30], and its functional traits show no significant correlation with NO_x_ levels. The significant positive correlation between its leaf δ^15^N value and atmospheric CO_2_ concentration is similar to that observed in *G. biloba*. Likewise, as with *S. japonica* and *G. biloba*, *P. tomentosa* exhibits a significant decline in IWUE with an increasing atmospheric CO_2_ concentration due to fertilization effects [40].

The relative abundance of symbiotic fungi in the root system of *P. tomentosa* is regulated by both soil nutrient availability and plant functional traits. The results indicate that fungal abundance declines significantly with an elevated soil nitrogen-to-phosphorus (N/P) ratio, suggesting that *P. tomentosa* depends heavily on symbiotic fungi for nitrogen acquisition [52], but its phosphorus uptake efficiency may be less constrained by fungal symbiosis. This may be due to the lack of a specialized phosphorus acquisition strategy, unlike *G. biloba*. Additionally, SLA was significantly negatively correlated with fungal abundance. This may reflect trade-offs in plant carbon allocation strategies: a high SLA is associated with a fast-growth strategy that demands continuous nutrient input [53], whereas low fungal abundance may indicate that more carbon is allocated to plant tissues rather than to mycorrhizal associations.

In summary, the relative abundance of symbiotic fungi in *P. tomentosa* roots is influenced by the soil nutrient stoichiometry (N/P ratio) and leaf economic strategy (SLA). An elevated soil N/P ratio reduces fungal abundance, reflecting the dependence of the plant on mycorrhizae for nitrogen nutrition when phosphorus is relatively abundant. Concurrently, a high SLA correlates with reduced fungal colonization, indicating a carbon allocation trade-off, in which investments in rapid leaf turnover may limit resources allocated to symbiotic maintenance.

## 5. Conclusions

*S. japonica*, *G. biloba*, and *P. tomentosa* exhibit distinct mechanisms of atmosphere–plant–soil interactions. *S. japonica*, with its capacity to assimilate atmospheric NO_x_, alters the internal nitrogen balance via changes in leaf δ^15^N, which in turn promotes the abundance of symbiotic fungi, demonstrating a clear cascade effect in plant–microbe interactions. In contrast, *G. biloba*, with limited NO_x_ assimilation capacity, shows a negative correlation between symbiotic fungal abundance and soil available nitrogen and phosphorus, highlighting its reliance on mycorrhizal networks for nutrient acquisition under poor soil conditions. For *P. tomentosa*, which also lacks the ability to assimilate atmospheric NO_x_, fungal abundance is negatively associated with the soil N/P ratio and SLA. This suggests that increased N/P weakens the dependence of plants on mycorrhizal nitrogen uptake, and a high SLA reflects a carbon allocation strategy that reduces investment in mycorrhizal symbiosis.

Based on the findings of this study, we propose the following recommendations: *S. japonica*, due to its strong potential for air pollution mitigation, should be further promoted as a street tree species. For *G. biloba*, symbiotic fungal abundance is limited by soil nitrogen and phosphorus, particularly phosphorus; thus, balanced fertilization, with an emphasis on phosphorus, is advised. In the case of *P. tomentosa*, symbiotic fungi are primarily influenced by the soil nitrogen-to-phosphorus ratio and atmospheric CO_2_, with nitrogen having a more pronounced effect. Therefore, it is recommended to apply nitrogen fertilizers appropriately or, preferably, adopt mixed planting with *S. japonica*, which can assimilate atmospheric NO_x_, to enhance overall ecosystem services.

## Data Availability

All of the data that support the findings of this study are available in the main text.

## Abbreviations

The following abbreviations are used in this manuscript:

GAM	Generalized additive model
SEM	Structural equation modeling
SLA	Specific leaf area
IWUE	Intrinsic water-use efficiency
LD	Leaf density
